# Comparative Evaluation of Various Temperature Changes on Stress Distribution in Class II Mesial-occlusal-distal Preparation restored with Different Restorative Materials: A Finite Element Analysis

**DOI:** 10.5005/jp-journals-10005-1505

**Published:** 2018-06-01

**Authors:** Binita Srivastava, Neorem N Devi, Nidhi Gupta, Rashi Singh

**Affiliations:** 1 Professor and Head, Department of Pedodontics and Preventive Dentistry, Santosh Dental College and Hospital, Ghaziabad, Uttar Pradesh, India; 2 Postgraduate Student, Department of Pedodontics and Preventive Dentistry, Santosh Dental College and Hospital, Ghaziabad, Uttar Pradesh, India; 3 Reader, Department of Pedodontics and Preventive Dentistry, Santosh Dental College and Hospital, Ghaziabad, Uttar Pradesh, India; 4 Senior Lecturer, Department of Pedodontics and Preventive Dentistry, Santosh Dental College and Hospital, Ghaziabad, Uttar Pradesh, India

**Keywords:** Finite element analysis, Maxillary second pre-molar, Mesial-occlusal-distal cavity, Thermal stress analysis.

## Abstract

**Introduction:**

The principal goal of dentistry is to maintain and improve the quality of life of the dental patients. As many of these objectives require the replacement or alterations of the existing tooth structure, the main challenge for centuries has been the development and selection of biocompatible materials that can withstand the unique conditions of the oral environment.

Finite element analysis (FEA) is a modern technique of numerical stress analysis that has become a solution to the task of predicting failure due to unknown stresses by showing problem areas in a material and allowing designers to see all of theoretical stresses within.

**Aims and objectives:**

To evaluate and compare the effect of various temperature changes on the stress distribution, in class II mesial-occlusal-distal (MOD) cavity when restored with different restorative materials, using the finite element method (FEM).

**Materials and methods:**

Using FEA, various thermal stresses generated in class II MOD lesion using different restorative materials were studied.

**Results:**

The computer-generated models of intact tooth and teeth restored with the different restorative materials were designed, and thermal stress at different temperatures was observed when subjected to the thermal loads of 5°C, 20°C, 36°C, and 55°C. From the results of the study, it can be concluded that glass ionomer cement (GIC) performed best, followed by intact tooth, composite resin, silver amalgam, and zinc oxide eugenol cement.

**Conclusion:**

Restoration of class II MOD lesions with materials of lower modulus of elasticity and lower coefficient of thermal expansion will enable better stress distribution.

**How to cite this article:** Srivastava B, Devi NN, Gupta N, Singh R. Comparative Evaluation of Various Temperature Changes on Stress Distribution in Class II Mesial-occlusal-distal Preparation restored with Different Restorative Materials: A Finite Element Analysis. Int J Clin Pediatr Dent 2018;11(3):167-170.

## INTRODUCTION

The main challenge for the centuries has been the development and selection of biocompatible materials that can withstand the unique conditions of the oral environment. In recent years, dentistry has witnessed the introduction and subsequent withdrawal of numerous unsatisfactorily materials and techniques from the markets, which do not comply with the oral conditions.^1^

With advancement and expansion in computation technology, FEA which originated during 1940s as a significant tool in the field of aeronautical engineering has been found to be of widespread use in the fields of medicine and dentistry to analyze the mechanical behavior of tooth structures and materials.2 It can be applied to solids of irregular geometry and heterogeneous material properties, making it suitable for the examination of structural behavior of teeth.^2^

The teeth always encounter different circumstances in the oral cavity, such as hot or cold liquids, solids, acidic materials, and different masticatory loads. Usually, thermal loads in the oral environment ranges from 0°C to 67°C.^3^ Due to change in temperature, dental restorations expand or contract more than the tooth, depending upon their thermal expansion coefficients, creating either tangential, tensile, or compressive stresses in the natural tooth. So far, there has been limited research carried out on the response of restorative materials when subjected to thermal stresses.^4^

There are generally two types of analysis that are used in the dentistry, i.e., two-dimensional and three-dimensional (3D) modeling.^2^ To determine the stress levels due to thermal and mechanical loads in a healthy and restored tooth, a thermomechanical FEA in 3D models is used.^5^

The ongoing search for a biocompatible material not only requires a material that has physio-mechanical properties similar to natural tooth structure, but in addition, has to be investigated from a thermal point of view to understand stresses generated by temperature changes in mouth.

The aim of this study was to analyze the temperature and thermal stresses distribution in an intact premo-lar tooth and teeth with a class II MOD cavity design, restored with different materials using the FEM.

**Fig. 1:**
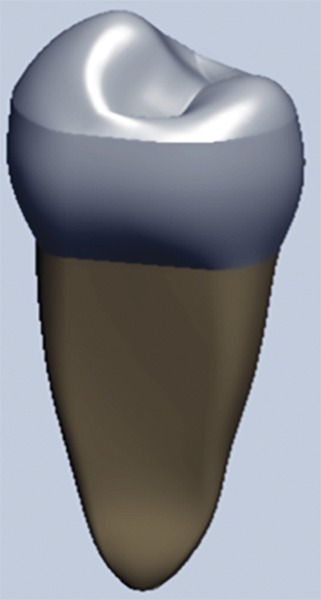
Solid virtual model of intact premolar tooth

**Fig. 2:**
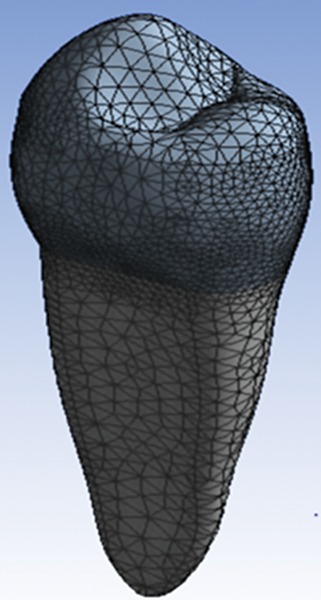
Final meshed finite element model of intact premolar tooth

**Fig. 3:**
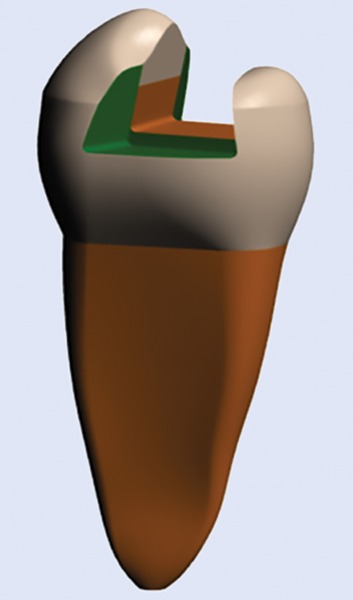
Solid virtual model of class II MOD cavity

## MATERIALS AND METHODS

An intact maxillary second premolar tooth was considered for this study. The reason being that a premolar tooth has a transitional shape and more complex geometry as compared with other teeth, and also it is one of the posterior teeth, thus can be analyzed for stress generation and load-bearing capacity.^3^

### Modeling of a Normal Premolar Tooth

The first step in FEA was modeling. The quality of the analysis results depends on the accuracy of the model. The tooth was subjected to a computed tomography (CT) scan and the cross-section of the tooth was obtained at an equal interval of 0.5 mm.

These sections were obtained in Digital Imaging and Communication of Medicine (DICOM) format and the data were fed into the computer. The DICOM is a neutral image format basically for medical imaging purposes like CT and magnetic resonance imaging (MRI).

Using the software Materialise Interactive Medical Image Control System (MIMICS), these cross-sections were converted into a 3D model. The MIMICS is an interaction tool for the visualization and segmentation of CT images as well as MRI images and 3D rendering of objects. Thus, a solid virtual model of the maxillary second premolar was obtained.

This model was exported to ANSYS software (Fig. 1).

### Meshing

The creation of the finite element model was divided into several finite elements. The element chosen for the study was tetrahedral, which is a 4-nodal element.

### Preparation of Virtual Cavity

With a computer-aided design (CAD) modeling program, the appropriate class II MOD cavity was designed in all the models.

After the cavity preparation, all the models were restored with different restorative materials.

The software ANSYS was used to create the finite element model of the same (Figs 2 and 3).

### Material Properties

The assignment of proper material problems to a finite element model is a necessary step to ensure predictive accuracy. Material properties of the tooth and the restorative materials were assigned to the models (Table 1).

**Table 1:** **Table 1:** Material properties assigned to the model

*Material*		*Young’s modulus, Mpa*		*Poisson’s ratio*		*Density, kg/m^3^*		*Specific heat, J/kg/°C*		*Thermal conductivity, W/m.k*		*Thermal expansion,1/°C × 10^-5^*	
Enamel		4.140E + 04		0.32		2800		750		0.84		10	
Dentin		1.860E + 04		0.3		2000		1302		0.63		11	
Composite resin		1.758E + 04		0.36		2000		820		1.26		34	
GIC		7.30E + 03		0.3		2100		750		1.5		10.5	
Siver amalgam		4.83E + 04		0.35		10500		240		23.1		25	
Zinc oxide eugenol		1.35E + 04		0.3		1600		548.471		1.67		35	

### Loading Conditions

The generated models, unrestored (intact tooth) and restored with composite resin, GIC, zinc oxide eugenol cement, and silver amalgam were then subjected to various thermal loads of 5°C, 20°C, 36°C, and 55°C.

Thermal loads were applied on the occlusal surface of premolar tooth for 15 seconds and the stress analysis was carried out using ANSYS solver.

### Restoration of the Lesion

- Model I: An intact maxillary premolar tooth.
- Model II: Maxillary premolar with class II MOD cavity design restored with composite resin (Kuraray, Clearfil AP-X).
- Model III: Maxillary premolar with class II MOD cavity design restored with GIC type II (3M ESPE, Ketac Molar).
- Model IV: Maxillary premolar with class II MOD cavity design restored with zinc oxide eugenol cement (DPI).
- Model V: Maxillary premolar with class II MOD cavity design restored with silver amalgam (Dentsply).

The stress distributions patterns were plotted using the general postprocessor of ANSYS.

## RESULTS

**Graph 1:**
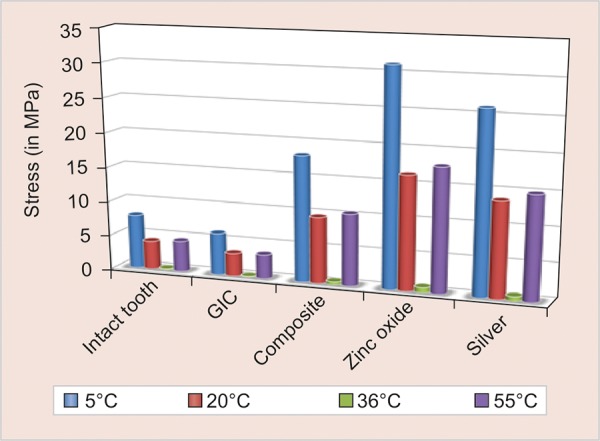
A comparison of the thermal stress values in intact tooth and in all the four restoration groups under different thermal loads

The thermal stresses in each of the models were studied. The results are displayed in terms of von Mises stress values (Table 2 and Graph 1).

## DISCUSSION

In the oral cavity, the tooth and the restoration are mainly subjected to two types of stresses, first, mechanical stress generated during functional and masticatory activities, and second, thermal stress due to temperature fluctuations. Hence, it is important to understand the pattern of stress distribution so as to enhance the longevity of the restorations.^6^

As reported by Palmer et al,^7^ thermal loads in the oral environment range between 0°C and 67°C.^5,8^ There are important parameters, such as thermal conductivity, thermal diffusivity, and linear coefficient of thermal expansion, which predict the transfer of thermal energy through a material during ingestion of hot and cold food and bever-ages.^5^ Thermal diffusivity of a material controls the time and rate of temperature change as heat passes through a material.^9^ It measures the rate at which a body with nonuni-form temperature reaches a state of thermal equilibrium. When dental structure is replaced with restorative materials, the thermal diffusivity of the tooth changes.^5^

The FEA is a new design concept which has the ability to accurately obtain the stress pattern throughout the structure under consideration, even if the structure is nonhomogeneous. Once a detailed CAD model has been developed, FEA can analyze the design in detail, reducing the amount of time and the number of prototypes required, thus rendering FEM a reliable and accurate method in biomechanical applications.^10^

**Table 2:** **Table 2:** Stress distribution patterns when thermal loads were applied

*Thermal loads*		*Intact tooth stress distribution in MPa*		*GIC stress distribution in MPa*		*Composite resin stress distribution in MPa*		*Silver amalgam in MPa*		*Zinc oxide eugenol cement in MPa*	
5°C		7.8496		6.0946		18.185		26.005		31.16	
20°C		4.1468		3.3196		9.6061		13.76		16.46	
36°C		0.19746		0.1533		0.45738		0.65628		0.783	
55°C		4.492		3.4874		10.404		14.959		17.82	

In the present study, thermal stresses distribution at different thermal loads on intact premolar tooth and class II MOD restored with different restorative materials were evaluated, using FEA. Four thermal loads applied were: 5°C (denotes the extreme cold temperature), 20°C (denotes cold beverages), 36°C (normal oral cavity temperature), and 55°C (denotes extreme hot temperature).

Results were displayed as color measurement bar in which each color corresponded to a range of stress values. Different shades of color indicate the amount of stress generated with dark red indicating maximum stress and dark blue indicating minimum stress.

At a temperature of 5°C, all the materials exhibited maximum stress distribution. The maximum stress generation was seen in ZOE cement (31.16 MPa) followed by silver amalgam (26.005 MPa), composite resin (18.185 MPa), and then GIC (6.0946 MPa).

However, the stress generation in GIC was less than that of intact tooth. This behavior of GIC and also other materials can be explained based on Young’s modulus of elasticity and coefficient of thermal expansion. Materials having higher modulus of elasticity exhibit more rigidity in comparison with other. But the coefficient of thermal expansion also plays an important role. Materials having low modulus of elasticity but higher coefficient of expansion exhibit higher thermal stresses.

As the temperature was further increased and thermal loads varied, the stress generation reduced with an increase in temperature. This was seen up to a temperature of 36°C which is assumed to be the normal oral cavity temperature and the tooth structure is considered to be stress-free at this temperature.^9,11^ With further increase in thermal load to hot temperature, the stress generation starts increasing with increase in temperature.

Thus, all materials function best at normal oral cavity temperature and there is increase in stress generation with any variation in thermal temperature, though the stress generation is less in higher temperatures as compared with cold temperatures.^9^ This may be due to further increase in the rigidity of tooth structure at colder temperatures.

## CONCLUSION

The following conclusions were drawn:

- The best thermal stress distribution with minimal stress values was seen when tooth was restored with GIC.
- At 36°C, intact tooth and all the restored teeth with different restorative materials showed minimum stress.
- When the tooth was loaded at 5°C, all restorative materials showed the maximum stress development.
- At 20°C and 55°C, all restorative materials showed a comparable thermal stress distribution.
- At 36°C, which is close to the oral cavity temperature, GIC withstands maximum stress and zinc oxide cement bears minimum stress.
- Both intact tooth and GIC restoration showed a comparable stress distribution pattern. Thus, GIC was found to be a better restorative material in distributing thermal stresses in the present study.

The FEM is the nearest possible method available today to simulate the oral cavity *in vitro.* It is a new design concept suitable for modeling asymmetrical structures with significant geometrical elements determining their real-world behavior under various load environments. The use of these theoretical engineering methods will certainly give answers to multiple problems in dentistry. Thus, the results are practical and applicable, of clinical significance and reference value, and give direction to experimental and clinical research.
